# Characterization of the complete mitochondrial genome of demersal flatfish *Eopsetta grigorjewi* (Pleuronectiformes: Pleuronectidae) from South Korea

**DOI:** 10.1080/23802359.2022.2072244

**Published:** 2022-05-11

**Authors:** Maheshkumar Prakash Patil, Jong-Oh Kim, Yong Bae Seo, Jiyoung Shin, Ji-Young Yang, Gun-Do Kim

**Affiliations:** a Industry-University Cooperation Foundation, Pukyong National University, Busan, Republic of Korea; b Department of Microbiology, Pukyong National University, Busan, Republic of Korea; c School of Marine and Fisheries Life Science, Pukyong National University, Busan, Republic of Korea; d Research Institute for Basic Science, Pukyong National University, Busan, Republic of Korea; e Institute of Food Science, Pukyong National University, Busan, Republic of Korea; f Department of Food Science and Technology, Pukyong National University, Busan, Republic of Korea

**Keywords:** *Eopsetta grigorjewi*, Pleuronectidae, mitochondrial genome, phylogenetic analysis

## Abstract

*Eopsetta grigorjewi* (Pleuronectiformes: Pleuronectidae) is a demersal flatfish found in South Korea, Japan, Taiwan, China, and the Yellow Sea. *E. grigorjewi* complete mitochondrion DNA (mtDNA) consists of 16,921 bp and a 54% A + T content. It includes 2 ribosomal RNA (rRNA), 22 transfer RNA (tRNA), 13 protein-coding genes, and 1 non-coding regulatory area. ND2, ND3, ND4, COII, COIII, ATPase6, and CytB all have incomplete stop codon genes. The evolutionary analysis of 13 species from the same family indicated a close relationship. This work will be valuable for future research on molecular evolution and the creation of biomarker databases for determining the originality of *E. grigorjewi*.

*Eopsetta grigorjewi* (Herzenstein, 1890) is a member of the Pleuronectiformes order, Pleuronectidae family, and Pleuronectinae subfamily (Froese and Pauly 2012). It is also known as mushigarei, round nose flounder, shothole flounder, and shotted halibut (Robins et al. 1991). It resides near the sublittoral zone at depths of 200–4000 feet, has an adult fish length of around 24 inches, and female fishes are bigger than male fishes (Kim et al.^2011^). These fish are East Asian marine fauna found in the Western Pacific Ocean, including the east coast of the Republic of Korea, the north coast of Japan, the south shore of Taiwan, China, and the Yellow Sea (Froese and Pauly 2018). Currently, the genus *Eopsetta* is known to include two species: *E. grigorjewi* and *E. jordani* Lokington, 1879. (Froese and Pauly 2012). Fish is high in protein, fatty acids, and minerals. The majority of Koreans consume seafood, and the eating culture is similar to that of Japan and China. People consume fish that has been cooked with seasonings, fried, or consumed raw. In the sea fish industry, flatfish are very important for export/import. It is critical to distinguish between imported and exported fish origins. In this study, we are focusing on characterizing mitochondrial DNA (mtDNA), which will aid in the development of molecular identification tools and will be a useful resource in the future to study genetic diversity and molecular evaluation.

The specimen with voucher specimen number MFDS-FGA12 was captured by a trawl net off the coast of Busan (East Sea), Republic of Korea (35°06′28.1″N 129°12′40.3″E). It was deposited at the Department of Food Engineering, Pukyong National University, Busan, Republic of Korea (Ji-Young Yang, jyyang@pknu.ac.kr). Following the manufacturer's recommendations, genomic DNA was produced using the DNeasy Blood and Tissue Kit, Qiagen, Germany. The DNA library was produced and sequenced on the MGISEQ-2000 system utilizing paired-end reads (150 bp) using the MGIEasy DNA Library Prep Kit (MGI, China). Cutadapt ver. 1.9 (Martin 2011) was used to clean the raw reads, and the cleaned data was mapped using CLC Genomics Workbench (Qiagen, Germany). The final mtDNA sequence was annotated using the MitoFish-database of the fish mitochondrial genome (Sato et al. 2018). The MEGA11 platform was used to construct a Pleuronectidae phylogenetic relationship tree using the maximum likelihood method and the Tamura-Nei model with 1000 bootstraps (Tamura et al. 2021).

The whole genome was sequenced using 32,704,409 read-pairs, yielding 9,811,322,700 bp. The whole mtDNA of *E. grigorjewi* is close-circular, with 16,921 bp deposited in GenBank (Accession number OK545542.1). It has a 54% A + T content, and the individual base-pair composition includes A 27% (4635 bp), T 27% (4309 bp), G 17% (2915 bp), and C 29% (5062 bp). Two ribosomal RNA (rRNA), twenty-two transfer RNA (tRNA), thirteen protein-coding genes, and one regulatory area are among the 38 components of *E. grigorjewi* mtDNA. Nine of these genes, notably ND6 and eight tRNAs, were found on the L-strand (Gln, Ala, Asn, Cys, Tyr, Ser, Glu, Pro). At the present, mtDNA has been shown to contain incomplete genes, including the stopping codons of ND2, ND3, ND4, COII, COIII, Cyt B, and ATPase 6, and atypical codon usage in tRNA-Ser (GCU, UGA) and tRNA-Leu (UAG, UAA).

Phylogenetic relationships tree within the Pleuronectidae family were built using a published mtDNA dataset, as shown in Figure 1. The phylogenetic study revealed that *E. grigorjewi* (Accession no. OK545542.1) is closely related to other genera and species in the Pleuronectidae family. For a better understanding of the species phylogeny, a detailed morphological and molecular phylogenetic investigation is required. These findings will help in understanding the genetic diversity and evolution of *Eopsetta* and Pleuronectidae. The present study's mtDNA results will be useful for future research on molecular evolution and the construction of a biomarkers database for determining the originality of *E. grigorjewi*.

**Figure 1.**
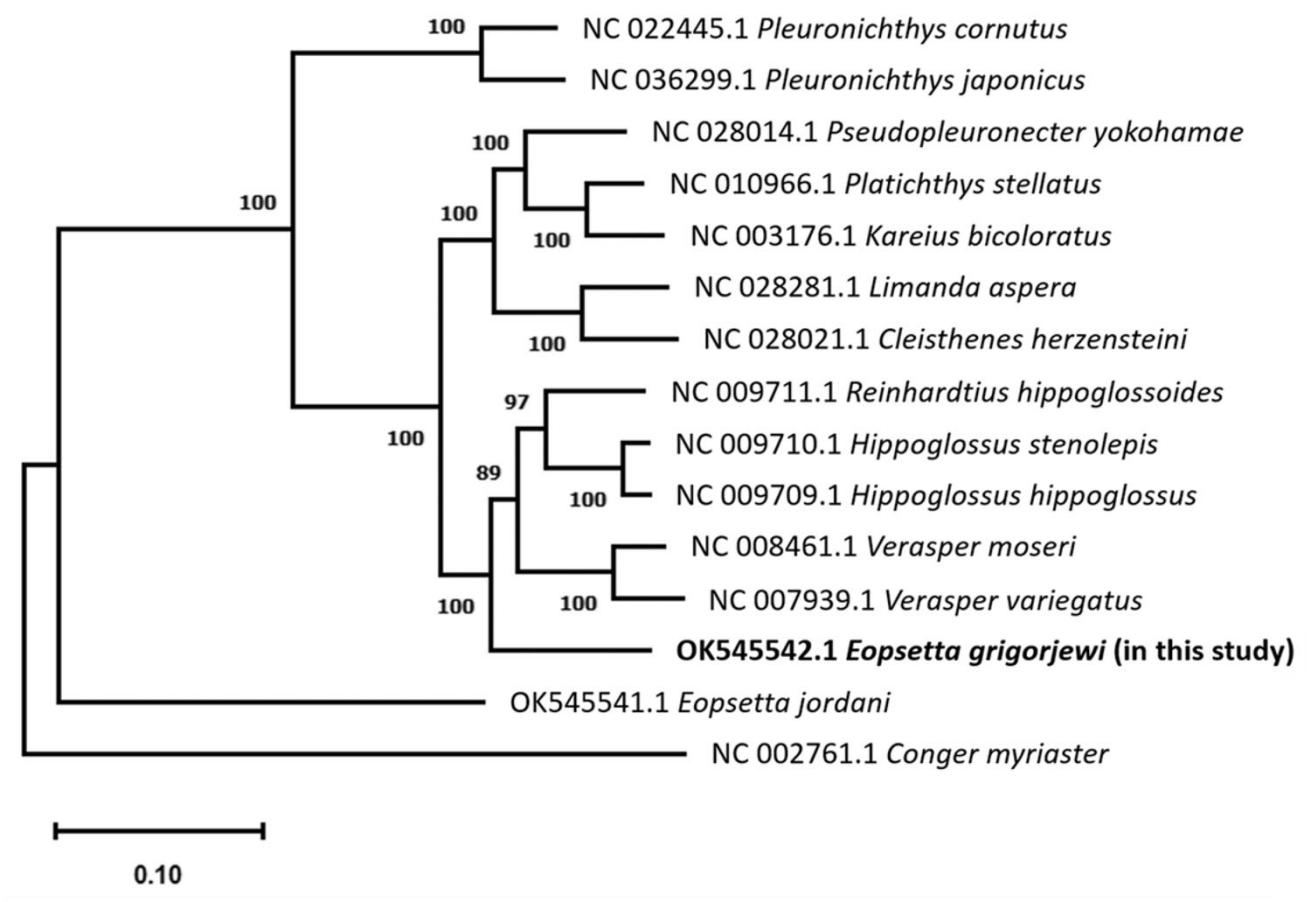
A phylogenetic tree within family Pleuronectidae, constructed with mtDNA data from NCBI GenBank of 13 species, *Eopsetta grigorjewi* (present study) and *Conger myriaster* was set as the outgroup using MEGA11 (ClustalW, maximum likelihood method and Tamaru-Nei model) with 1000 bootstrap replicates. Bootstrap possibilities are indicated by numbers on each node.

## Ethical approval

The sample used for this study was a dead body of fish and as per the animal experimental ethics in the Republic of Korea (Standard operating guideline; IACUC – Institutional Animal Care and Use Committee, Book no. 11-1543061-000457-01, effective from Dec. 2020) does not need any approval from Ethics Committee.

## Data Availability

The genome sequence data that support the findings of this study are openly available in GenBank of NCBI at (https://www.ncbi.nlm.nih.gov/) under the accession no. OK545542.1. The associated BioProject, BioSample, and SRA numbers are PRJNA794315, SAMN24624474, and SRP353559, respectively.
